# Estrogenic Regulation of Histamine Receptor Subtype H1 Expression in the Ventromedial Nucleus of the Hypothalamus in Female Rats

**DOI:** 10.1371/journal.pone.0096232

**Published:** 2014-05-07

**Authors:** Hiroko Mori, Ken-Ichi Matsuda, Masanaga Yamawaki, Mitsuhiro Kawata

**Affiliations:** 1 Department of Medical Education, Kyoto Prefectural University of Medicine, Kawaramachi Hirokoji, Kamigyo-ku, Kyoto, Japan; 2 Department of Anatomy and Neurobiology, Kyoto Prefectural University of Medicine, Kawaramachi Hirokoji, Kamigyo-ku, Kyoto, Japan; University of Rouen, France

## Abstract

Female sexual behavior is controlled by central estrogenic action in the ventromedial nucleus of the hypothalamus (VMN). This region plays a pivotal role in facilitating sex-related behavior in response to estrogen stimulation via neural activation by several neurotransmitters, including histamine, which participates in this mechanism through its strong neural potentiating action. However, the mechanism through which estrogen signaling is linked to the histamine system in the VMN is unclear. This study was undertaken to investigate the relationship between estrogen and histamine receptor subtype H1 (H1R), which is a potent subtype among histamine receptors in the brain. We show localization of H1R exclusively in the ventrolateral subregion of the female VMN (vl VMN), and not in the dorsomedial subregion. In the vl VMN, abundantly expressed H1R were mostly colocalized with estrogen receptor α. Intriguingly, H1R mRNA levels in the vl VMN were significantly elevated in ovariectomized female rats treated with estrogen benzoate. These data suggest that estrogen can amplify histamine signaling by enhancing H1R expression in the vl VMN. This enhancement of histamine signaling might be functionally important for allowing neural excitation in response to estrogen stimulation of the neural circuit and may serve as an accelerator of female sexual arousal.

## Introduction

The ventromedial nucleus of the hypothalamus (VMN) is a brain region governing female reproductive functions and sexual behavior [1]–[4], and is anatomically divided into the ventrolateral (vl) and dorsomedial (dm) subregions. Induction of female sexual behavior is predominantly controlled by sex steroid hormonal action in the accompanying triggering of sexual arousal [1], [5], [6]. Estrous females with elevated serum estrogen show sexual responsiveness with high neural arousal. Central estrogen affects the estrogen-sensitive neural group in the VMN, and particularly in the vl VMN and thus the neural circuit controlling female sexual behavior is “turned on”. This primary estrogenic action is thought to be a genomic effect mediated by estrogen receptor (ER) [7], which is abundantly present in the vl VMN [1], [8], [9].

Histamine is a potent arousal-related neurotransmitter that typically excites neurons by causing depolarization, which increases firing latency [10]–[12]. Histaminergic neurons are exclusively present in the tuberomammillary nucleus (TM) of the hypothalamus, from where they send projections to the whole brain, with the basal hypothalamic areas, including the VMN, receiving strong innervation [10], [11]. Histamine is synthesized by a specific enzyme, histidine decarboxylase, in the TM, and acts through selective receptors. Among the three well-established subtypes of histamine receptors; H1R, H2R and H3R in the vertebrate brain [11], [13], H1R subtype mainly mediates excitability effects of histamine by causing large depolarization and increased firing rates in most brain regions [10], [11], [14]–[17], while H2R and H3R are autoreceptors that inhibit synthesis and release of histamine [13], [18], [19]. The histamine action via H1R has been thought to be involved in estrogen-induced copulatory responsiveness in female rats [14], [15], that is interfered with by H1R blockage with injection of pyrilamine into the lateral ventricle [20]. However the detailed mechanism mediating estrogen signaling to the histamine system in VMN-related arousal is not fully understood. Our finding of colocalization of ERα and H1R in the vl VMN led us to hypothesize that there could be a functional interaction between estrogen signaling and histamine receptors.

In this study, we examined the regional distribution of H1R in the VMN and the changes in H1R expression level induced by hormonal manipulation in ovariectomized (OVX) females. A double immunofluorescence study showed localization of H1R in the vl VMN, exclusively coexisting with abundant ERα. This coexistence of ERα and H1R in neurons in the vl VMN implies a specific and functional interaction between estrogen signaling and the neural histamine system. Consistent with this, estrogen signaling acts as a principal regulator in VMN-mediated arousal by controlling several neuroendocrine factors in behavioral facilitation [1]–[5], [9].

We also found that estrogenic stimulation affects H1R expression levels in the female vl VMN. H1R mRNA levels were significantly increased in OVX female rats treated with estradiol benzoate (EB). In the dm VMN, there was no estrogenic effect on H1R expression. These observations suggest that a key aspect of the VMN-related sexual arousal mechanism involves estrogen amplifying neural sensitivity to histamine by inducing H1R expression in vl VMN neurons and maintaining a state of heightened neural arousal during hormonal stimulation.

## Materials and Methods

### Animals

Adult male and female Sprague-Dawley rats (Shimizu Laboratory Supplies Co.) were housed in cages with standard bedding and with water and food ad libitum. The temperature of the colony was maintained at 22°C with a 12:12 light:dark cycle (06:00 lights on, 18:00 lights off).

### Ethics Statement

The experiments were conducted in accordance with the Rules and Regulations of Animal Research, Kyoto Prefectural University of Medicine and were approved by the Ethics Committee for Animal Experiments at the Institute of Kyoto Prefectural University of Medicine. Approval ID: M21-110.

### Ovariectomy (OVX)

Female rats (8 weeks) were deeply anesthetized with 2% isoflurane and sodium pentobarbital (100 mg/kg i.p.) and bilateral ovaries were removed [21]. The rats were used in experiments 3 weeks after OVX.

### Hormonal manipulation in OVX female rats

OVX females were s.c. injected with estradiol benzoate (EB, 5 µg/100 µl of sesame oil, n = 30) or 100 µl sesame oil (n = 10) at noon 3 weeks post-OVX and sacrificed 24 h (n = 10) or 48 h (n = 10) after EB injection. Ten OVX females were s.c. injected with EB and progesterone (500 µg/100 µl of sesame oil) 48 and 4 h, respectively before the sampling. At designated times tissues from the rats were used for Western blot and real time PCR analyses.

### Tissue preparations

For immunohistochemistry, the rats were deeply anesthetized with 2% isoflurane followed by a lethal dose of sodium pentobarbital (150 mg/kg i.p.) and perfused intracardially with 0.9% NaCl followed by 4% paraformaldehyde (PFA) in 0.1 M phosphate buffer saline (PBS) (pH 7.4). The brains were immediately removed, postfixed in 4% PFA overnight at 4°C, and then submerged in 25% sucrose in 0.1 M PBS for 2 days. Coronal sections containing the VMN were cut on a cryostat (CM 3050, Leica) into a series of 40 µm thick sections for immunohistochemical studies.

### Sampling of VMN subregion tissues

**Figure 1:**
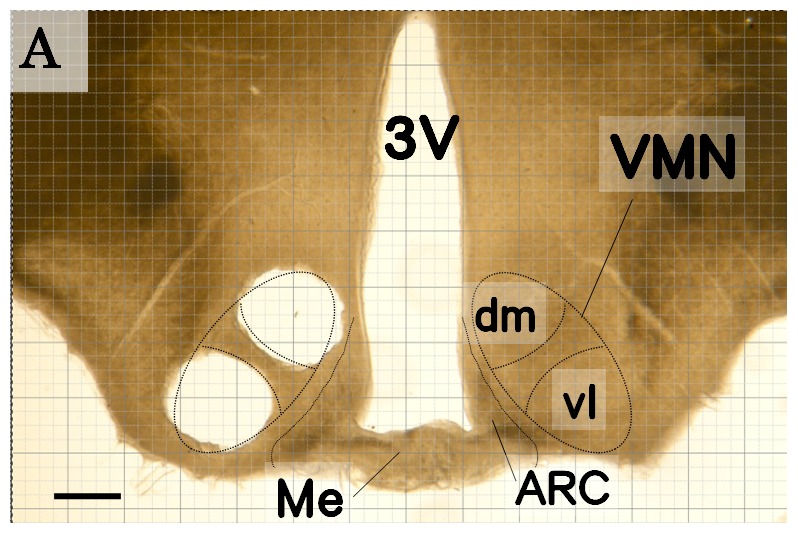
Sampling of VMN subregion tissues. Quantitative analysis of H1R mRNA and H1R protein in the two subregions of the VMN was performed after selective dissection of the vl VMN and dm VMN using a stainless steel needle. The dotted lines show the locations of the VMN and vl and dm subregions in the rat basal hypothalamus. (A) Representative photograph of a coronal brain section after sampling (left side). The vl VMN and dm VMN were selectively dissected with the needle (inner diameter 900 µm). An outline of the rat basal hypothalamus used for identification and selective dissection of the VMN subregions. 3V, Third ventricle; VMN, ventromedial nucleus of the hypothalamus; vl, ventrolateral part of the VMN; dm, dorsomedial part of the VMN; Me, Median eminence; ARC, arcuate nucleus. Scale bars, 500 µm.

For Western blot and real-time PCR, whole brains were removed under anesthesia (2% isoflurane and a lethal dose of sodium pentobarbital, 150 mg/kg body weight. i.p.). To obtain vl VMN and dm VMN tissues selectively, the brains were cut into 500 µm thick coronal sections using a microvibratome (D.S.K. Microslicer DTK-300W; Dosaka EM, Kyoto, Japan). Two serial sections containing the entire VMN subregion were mounted on glass, and the vl VMN or dm VMN region was dissected bilaterally using a stainless steel needle (inner diameter 900 µm) under a stereomicroscope (Fig. 1)[8]. Rat brain maps and preliminary Nissl staining and ERα immunostaining were used to identify the VMN subregions (Fig. 1). All dissected tissues were snap-frozen in liquid nitrogen.

### Immunohistochemistry

Brains of female rats were used in the colocalization study (n = 5), propidium iodide (PI) staining and preabsorption tests (n = 4). For colocalization studies using confocal microscopy, brain sections were blocked with 2% BSA in PBS with 0.3% Triton-X for 2 h at room temperature. Primary antibody incubations were performed using a mixture of rabbit polyclonal antibody to ERα (C1355; Millipore, Billerica, MA; 1∶1,000 dilution) and goat polyclonal antibody to H1R (LS-B1745; Life Span Bio Science, Inc.; 1∶1,000 dilution) in blocking solution overnight at 4°C. These sections were then washed with PBS three times and incubated for 1.5 h with both Alexa Fluor 488 anti-rabbit IgG (Molecular Probes, Inc., Eugene, OR) and Alexa Fluor 546 anti-goat IgG (Molecular Probes). Several sections were counterstained with PI (1 µg/ml) to visualize the nucleus. After washing with 0.1 M PBS, the sections were mounted and observed with a confocal laser scanning microscope (LSM 510META, Carl Zeiss, Jena, Germany). The specificity of the H1R antibody (LS-B1745; Life Span Bio Science, Inc.) was confirmed in a preabsorption test with control peptide. Detection of H1R immunoreactivity was carried out by using biotin/peroxidase conjugated streptavidin amplification system (Histofine SAB-PO kit, Nichirei) with diaminobenzidine used as the substrate [21]. Furthermore the specificity of LS-B1745 was also confirmed in cell line overexpressing H1R and in H1R KO mice (Hrh1 KO/C75BL/6) [22] (refer to File S1 and File S2 for details).

### Western blot analysis

Brain tissues of the vl VMN and dm VMN were lysed with 30 µl of sample buffer. After boiling for 5 min, the lysates (10 µl for H1R detection, 4 µl for tubulin detection) were subjected to 7.5% SDS-PAGE. To normalize the starting materials, tubulin was used as a loading control. Samples were electroblotted onto a polyvinylidene difluoride membrane (Millipore, Bedford, MA) using a semi-dry blotting apparatus (Bio-Rad Laboratories, Hercules, CA). The blotted membranes were blocked with 1.5% skim milk TBST at 4°C for 20 min and then incubated with rabbit polyclonal H1R antibodies (AHR-001; Alomone Labs, Jerusalem, Israel; 1∶1,000 dilution) and mouse monoclonal antibody to tubulin (T5201; Sigma Japan, Tokyo, Japan;1∶30,000 dilution in TBST) at 4°C for 12 h. The specificity of the AHR-001 antibody was shown in a preabsorption test with control peptide antigen (see product information for AHR-001; Alomone Labs, Jerusalem, Israel). Blots were washed three times with TBST and incubated with alkaline phosphatase-conjugated anti-IgG secondary antibody (Chemicon, Temecula, CA; 1∶1,000 dilution in TBST) for 2 h at room temperature. After washing three times with TBST, the blots were visualized using a nitroblue tetrazolium and 5-bromo-4-chloro-3-indolyl phosphate staining kit (Nacalai Tesque, Kyoto, Japan). Results were quantified by densitometric analysis using ImageJ software (National Institutes of Health, Bethesda, MD). Data are expressed as the ratios of H1R protein to tubulin protein in the vl VMN and dm VMN.

### Quantitative real-time PCR

Total RNA was extracted using an RNeasy Micro Kit (Qiagen, Hilden, Germany) from dissected vl VMN and dm VMN tissues and reverse-transcribed using ReverTra Ace (Toyobo, Osaka, Japan) and random hexamers. Gene expression was assessed by quantitative real-time PCR using a Light Cycler 480 II with Universal Probe Library assays (Roche Diagnostics, Mannheim, Germany). Reactions were performed in duplicate in 96-well plates for 55 amplification cycles (95°C, 10 s; 60°C, 10 s; 72°C, 10 s) in a 10-µl reaction volume. Primers were designed using the Roche Universal Probe Library Assay Design Center (www.universalprobelibrary.com): H1R forward primer: CTG TTT CCG TCT CGA CAT CA; H1R reverse primer: TGA GCC CCT GAC ATC TCC, and glyceraldehyde-3-phosphate dehydrogenase (GAPDH) forward primer: ACA ACT TTG GCA TCG TGG A; GAPDH reverse primer: CTT CTG AGT GGC AGT GAT GG. The carboxyfluorescein-labeled probe (Universal Probe Library, Roche Diagnostics) numbers were 5 for H1R and 114 for GAPDH. Data were normalized based on expression of the GAPDH gene. For quantification of changes in gene expression, the comparative C_p_ (threshold cycle number) method was applied to calculate the relative fold changes normalized against GAPDH.

### Statistical analysis

All values are shown as the group mean ±SEM. The significance of differences in H1R mRNA and H1R protein levels in the VMN for sex and female hormonal status were analyzed by two-way ANOVA (effects of sex and hormonal status) followed by a Tukey-Kramer *post hoc* analysis (multiple tests) for data from Western blot analysis and quantitative real-time PCR, with *P*<0.05 considered statistically significant.

## Results

### H1R localization in the ventrolateral VMN

**Figure 2:**
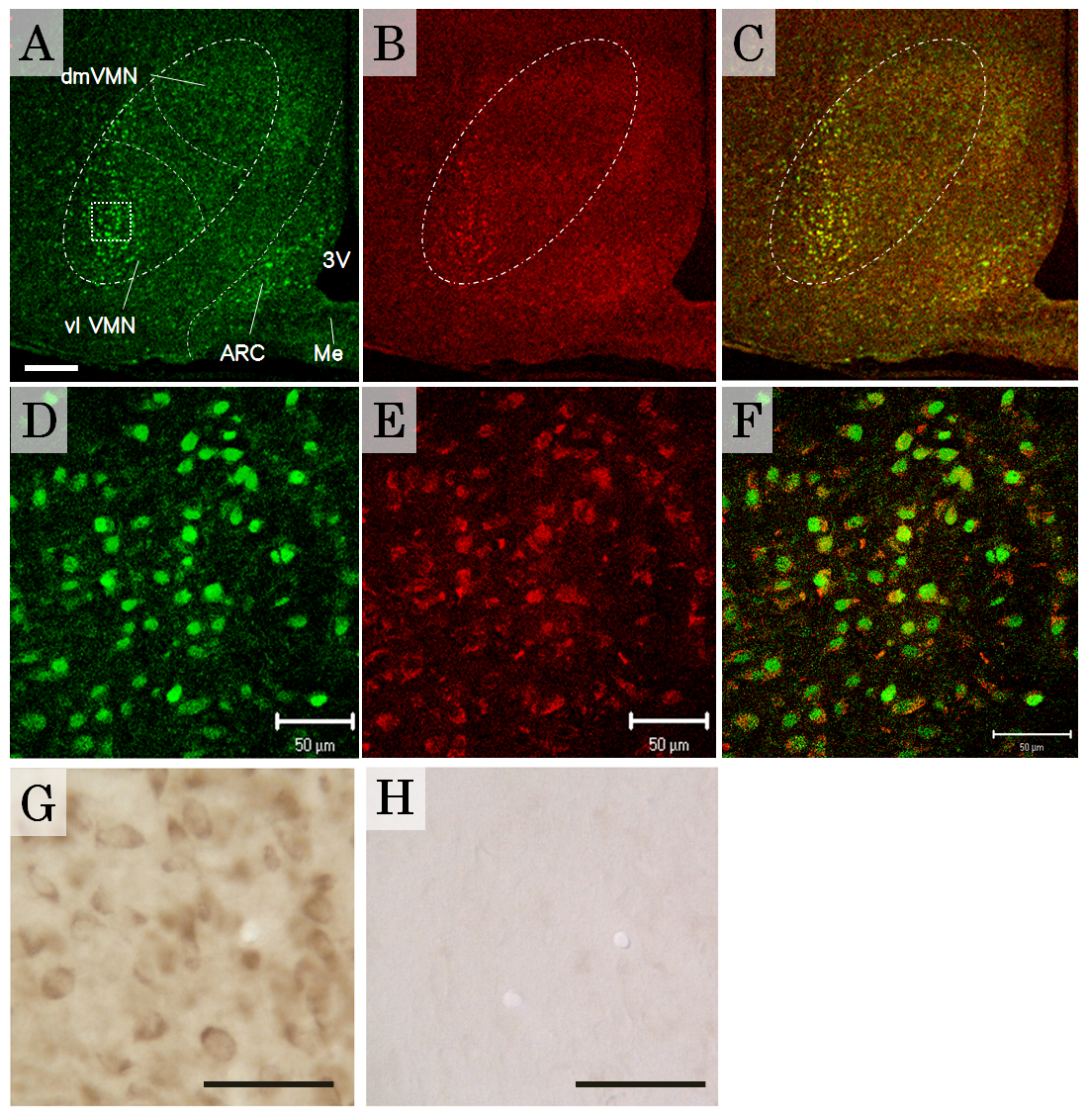
Distribution of H1R in the rat VMN. (A–F) Confocal microscopy images of dual immunofluorescence labeling of ERα (green, A and D) and H1R (red, B and E) in adult female rats. ERα-ir neurons are abundant in the vl VMN, but few are present in the dm VMN (A). (B) The highest density of H1R expression was found in the vl VMN. Merged images showed the coexistence of abundant ERα-ir and H1R-ir in the VMN (C and F). (D–F) Medium magnification images of representative neural groups within the vl VMN (area with a dotted line), allowing identification of cellular localization of ERα and H1R. (G and H) Detection of H1R immunoreactivities in VMN with (H) or without (G) control peptide. Preabsorption of H1R antibody with its peptide eliminated H1R immunoreactivities (H). 3V, Third ventricle; VMN, ventromedial nucleus of the hypothalamus; vl, ventrolateral part of the VMN; dm, dorsomedial part of the VMN; Me, median eminence; ARC, arcuate nucleus. Scale bars, 500 µm (A–C), 50 µm (D–H).

**Figure 3:**
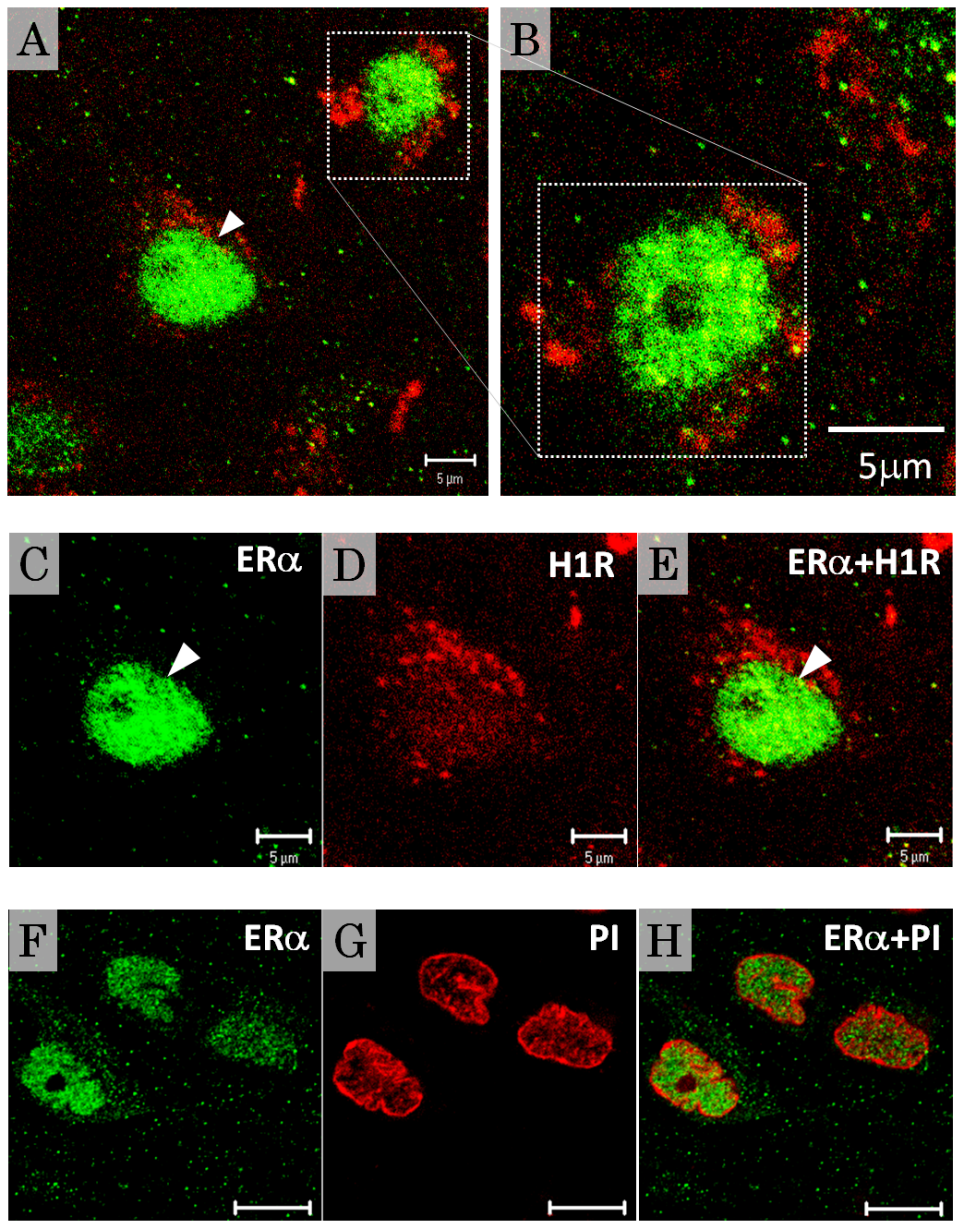
Subcellular localization of ERα and H1R in vl VMN neurons. (A, B, C, D, E) Representative images of dual immunofluorescence labeling of ERα (green) and H1R (red), obtained by confocal microscopy. Higher magnification images of individual cells in panel A are shown in B (area with a dotted line), and C, D and E (arrowheads). C–E are confocal images showing that ERα is exclusively localized in nuclei (green) and excluded from nucleoli (dark spot), while H1R is localized in the cell membrane (red). Panel E is a merge of the images from panels C and D. (F, G, H) Visualization of the cellular localization of ERα-ir in VMN neurons. ERα immunostaining (green) were observed in the nucleus defined by PI staining (red). Panel H is the merged image for panel F and G. ERα; ERα immunoreactivities, PI; propidium iodide. Scale bars, 5 µm (A–E), 10 µm (F–H).

The mediobasal hypothalamic area is densely innervated by histaminergic nerve terminals that originate from the TM nucleus. Histamine synthesized in TM neurons is transmitted via efferent fibers and acts at the target site through its receptor [10], [11]. In the present experiments, immunohistochemical staining showed region-specific localization of H1R in the rat mediobasal hypothalamic area (Fig. 2). The VMN and part of the arcuate nucleus displayed strong or moderate H1R immunoreactivity (-ir), as well as ERα-ir with region-related variations. Within the VMN, the distribution pattern of H1R was different in the vl VMN vs. the dm VMN. Within the VMN, the highest density of H1R-ir neurons was found in the vl VMN, with some H1R-ir in the dm VMN. Even within the dm VMN, a few neurons stained positively, suggesting that some neurons had a high level of H1R. Double immunofluorescence labeling of H1R and ERα showed specific colocalization, with both proteins expressed in almost all neurons in the vl VMN, but not in the dm VMN (Fig. 2). To confirm the specificity of the H1R antibody, a preabsorption test was performed with the blocking peptide LS-PB1745. Preabsorption of the antibody with the control peptide results in a loss of immunoreactivities (Fig. 2G and H). Importantly, almost all the distribution patterns of H1R-ir observed in the hypothalamus correspond to that of H1R mRNA in receptor autoradiography [23]–[25]. Furthermore, the specificity of the LS-1745 antibody was determined by showing no reaction in a negative control (H1R KO mouse: Figure S1 in File S1) and a specific reaction with H1R in a positive control (cell line overexpressing H1R: Figure S2 in File S2). Confocal microscopic images at high magnification showed the subcellular localization of the two receptors, with the nuclear receptor ERα preferentially located within nuclei and excluded from nucleoli (green fluorescence: Fig. 3A, B, C, E) and the G protein-coupled receptor H1R exclusively located in the cell membrane (red fluorescence: Fig. 3 A, B, D, E). The detailed localization of ERα and H1R is shown in Figs. 3 C and D, respectively, and the merged image is shown in Fig. 3E. Further, double staining with ERα and PI allowed direct observation of the intranuclear localization of ERα (Fig. 3 F, G, H). H1R-ir and ERα distribution patterns in the VMN were consistent across brain samples from 5 female rats, including in the preliminary experiments.

### Estrogenic regulation of H1R mRNA expression in the female VMN

Experiments were designed to study the effects of sex steroid treatment on H1R mRNA levels in the VMN of OVX female rats. Since the VMN exhibits sex-related differences in function and structure, we also examined H1R expression levels in males as a reference.

**Figure 4:**
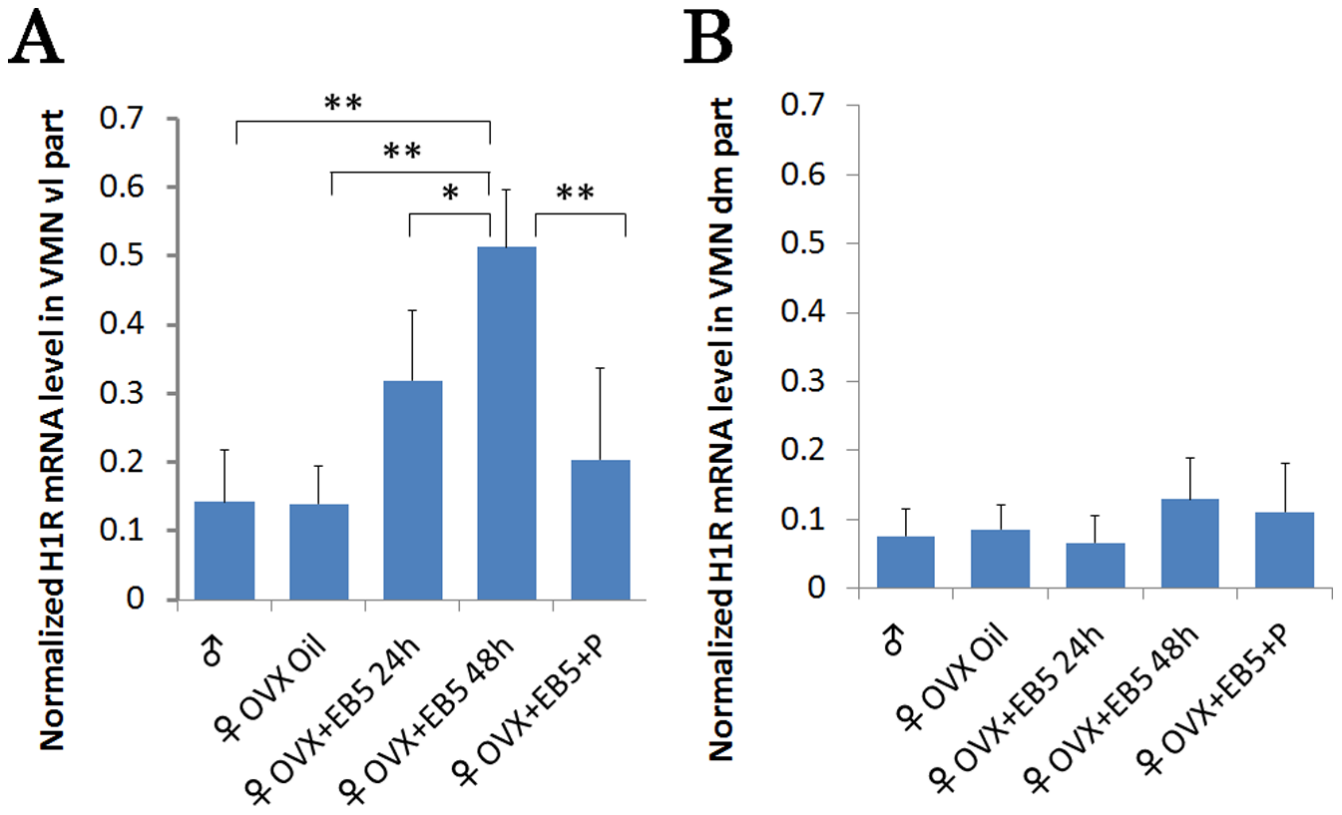
Quantitative evaluation of normalized H1R mRNA expression in the vl VMN and dm VMN. Normalized H1R mRNA expression levels were quantified for males (n = 5), OVX females (oil injected controls, n = 5), OVX females with EB (5 µg EB, 24 h or 48 h post-injection, n = 5 each), and OVX females with EB + progesterone (5 µg EB, followed by 500 µg progesterone, n = 5). In the vl VMN, H1R mRNA was significantly increased by EB in OVX females and decreased by progesterone. In the dm VMN, hormonal manipulation did not affect H1R mRNA expression. Data represents the absolute quantities of H1R mRNA compared with GAPDH mRNA amount. Data are presented as the mean ±SEM. **, P<0.01; *, P<0.05.

Quantitative real-time PCR of microscopically dissected samples from the vl VMN and dm VMN showed significantly elevated H1R mRNA in the vl VMN in EB-treated females. An approximately 5-fold increase in H1R mRNA was detected in EB (5 µg)-treated OVX females (48 h post-injection) compared with oil-treated OVX females (mean values ±0.51 vs. ±0.13, p<0.0001). Interestingly, the EB-induced increase in H1R mRNA was eliminated by progesterone injection (500 µg) after EB treatment. The H1R mRNA level differed between males and EB-injected OVX females, but not between males and oil-treated or progesterone-treated OVX females (Fig. 4A). In the dm VMN, there was no estrogenic effect on H1R mRNA in any group (Fig. 4B), showing the regional difference in estrogen responsiveness between the vl and dm regions of the VMN.

### Estrogenic regulation of H1R protein expression in the female VMN

**Figure 5:**
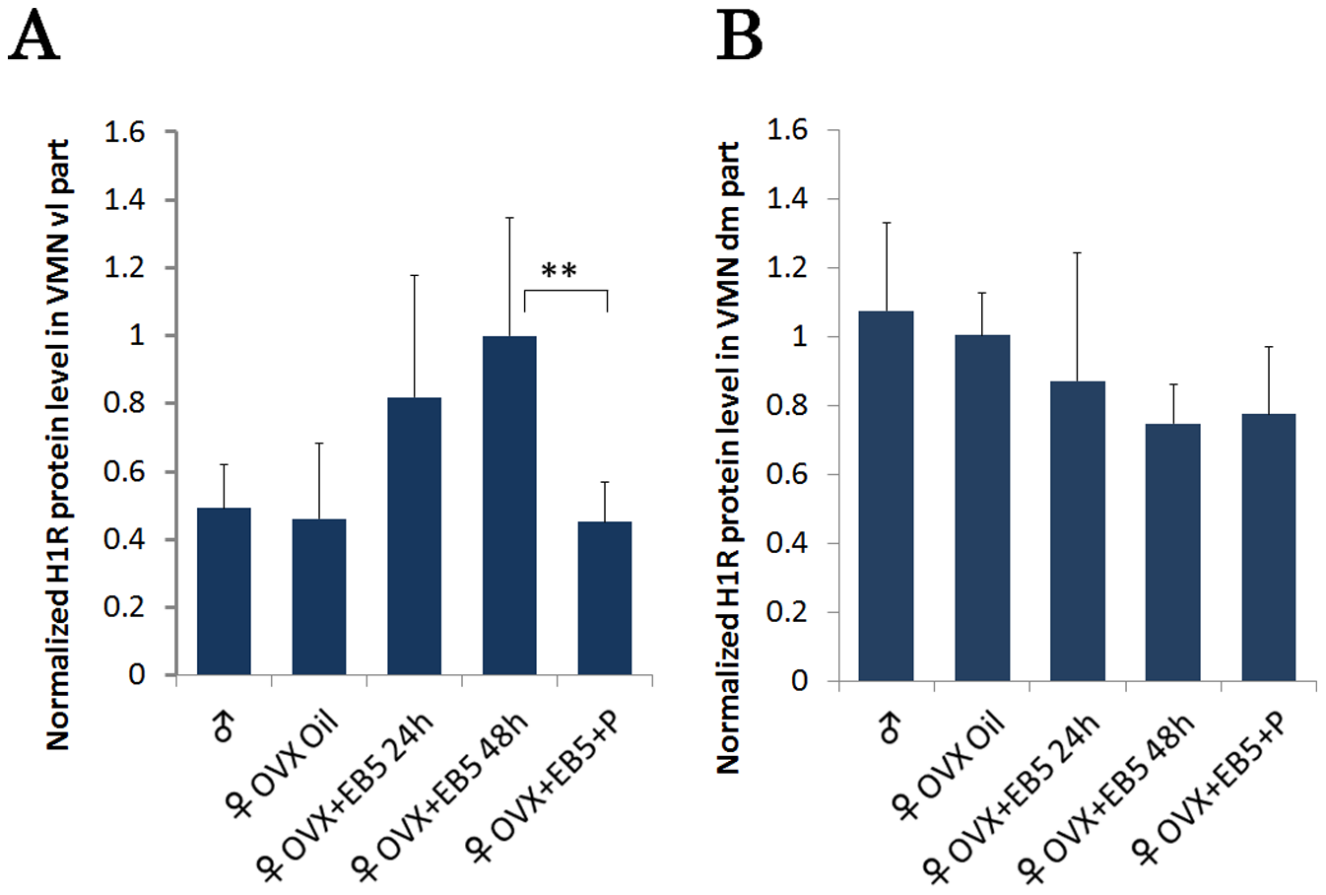
Quantitative evaluation of normalized H1R protein expression in the vl VMN and dm VMN. H1R protein expression levels were quantified for males (n = 5), OVX females (oil injected controls, n = 5), OVX females with EB (5 µg EB, 24 h or 48 h post-injection, n = 5 each), and OVX females with EB+ progesterone (5 µg EB, followed by 500 µg progesterone, n = 5). In the vl VMN, the H1R protein level tended to be increased by EB in OVX females (not statistically significant) and decreased by progesterone. In the dm VMN, hormonal manipulation did not affect H1R protein expression. Data are presented as the mean ±SEM. **, P<0.01. Data are expressed as the ratios of H1R protein to tubulin protein in the vl VMN and dm VMN regions. Since the immunoblotting were obtained independently for each region (from the respective immunoblotting membranes for the vl VMN and dm VMN), data were not to be compared between the two regions.

Next, we investigated the estrogenic effect on H1R protein expression in the vl VMN and dm VMN by Western blot analysis using tissues dissected in the same manner as those in the quantitative real-time PCR study. Anti-tubulin immunoblotting was performed simultaneously as a lording control to normalize the protein homogenate loading. In the vl VMN, H1R protein expression tended to be greater in EB (5 µg)-treated OVX females (24 h and48 h post-injection) than in control females, but the difference was not significant. H1R protein expression in EB treated OVX females was suppressed by subsequent injection of 500 µg progesterone (Fig. 5A). There was no difference in H1R protein expression in the vl VMN between males and control females. Consistent with the PCR results, there was no significant difference in H1R protein expression in the dm VMN among the groups (Fig. 5B). In these experiments, an immunopositive band was detected without additional bands, indicating that the antibody was indeed capable of recognizing H1R protein.

## Discussion

The objectives of this study were 1) to determine the regional distribution of H1R, 2) to investigate if H1R colocalizes with ERα, and 3) to test the hypothesis that sex steroids alter H1R expression levels in the female VMN, which is the principal regulator of arousal in female reproductive function. The neural histamine system contributes to arousal functions, including female sexual arousal, as shown in behavioral pharmacology experiments [20], [26]. However, the subnuclear localization of H1R in the VMN region has not been investigated. Thus, this is the first report describing the detailed localization of H1R in the VMN and the changes induced by sex steroids.

In the first set of experiments, we compared the H1R patterns in the female vl VMN and dm VMN, which are predicted to differ based on the generally accepted idea that these two regions have distinct regulatory roles in female sexual behavior [1], [27]. In support of our hypothesis, we found that the characters of H1R expressing neurons differed in the vl VMN and dm VMN, with coexistence of ERα and H1R in vl VMN neurons. Confocal microscopy revealed the exact subcellular colocalization of H1R and ERα in individual vl VMN neurons, with H1R in the cell membrane and ERα in the nucleus.

In the second part of the study, we determined whether exposure to sex steroids regulates H1R expression in the VMN, as predicted by the hypothesis for the effect of estrogen action on gene expression. To test the respective roles of sex steroids in the neural histamine system, we compared H1R expression levels among OVX females, OVX EB-treated females with and without progesterone, and male rats. PCR analysis revealed that H1R mRNA in the vl VMN appears to be regulated by sex steroids, since it was significantly induced in OVX EB-treated female rats. This effect was reversed by subsequent progesterone treatment. This hormonal regulation of H1R mRNA was specific to the vl VMN; there was no estrogenic effect on H1R in the dm VMN. This regional difference in the hormonal responsiveness of H1R between the vl VMN and dm VMN may be due to differential distribution of ERα in the two VMN subregions. ERα is a potent gene transcription factor that acts through genomic and non-genomic pathways upon ligand binding and is often coexpressed in neurons in which sex steroids cause changes in expression of a particular gene [28], [29]. Regulation of receptor protein expression by ligand-bound ERα has been well characterized for the progesterone receptor (PR) [30], [31] and oxytocin receptor [32], [33] in the VMN, and for vasopressin receptors [34], mu-opioid receptors [35]–[37], and cholecystokinin receptors [38]. In many cases, the changes in gene expression induced by estrogen have been suggested to be important for regulation of female sexual behavior, but they may also be linked to a wide range of sex-related functions. Importantly, in our study, a structural sex difference in terms of H1R expression emerged when OVX female rats were treated with EB, implying the importance of H1R in the VMN for sex-related functions.

Several studies have examined sexual differences in the rat neural histamine system. For example, the density of H1R in the cortex is higher in females than in males [39] and stress-related hypothalamic histamine release is higher in males than in females [40]. The suppressive effect of histidine on food intake has been found to be greater in females than in males, and this may be dependent on the presence of female sex steroids, since the effect of histidine was low in OVX females [41]. Sex-dependent differences in response to drugs that affect the neural histamine system have also been demonstrated in mice. Behavioral and pharmacological studies have shown that female mice are more sensitive to the arousal-reducing effects of H1R blocking agents in sensory stimuli responsiveness and running wheel activities [26]. These sex differences in histaminergic activities in the central nervous system may be relevant to some of the functional and behavioral differences between the sexes.

The neural histamine system may also be involved in facilitating female sexual behavior in rats. First, depletion of histamine by intraventricular administration of α-hydrazinohistidine, a histamine synthesis inhibitor, attenuates sexual behavioral performance in estrogen/progesterone-treated OVX female rats [20]. Second, intraventricular administration of H1R blocking agents (pyrilamine and chlorpheniramine) affects sexual behavior patterns with a marked decrease in lordotic quotient and with slight decrease in sexual receptivity in steroids-primed OVX females, suggesting the importance of H1R in maximal exertion of female sexual behaviors. [20]. However, this study used blockage of H1R by intraventricular administration of inhibitors, which affects a wide range of brain regions, rather than the VMN alone. Thus, despite central histamine signaling being understood to be important for steroid-induced sexual arousal in females, there is a lack of neuroanatomical data. In this situation, the local effects of histaminergic inputs in the VMN neural group on the behavior and detailed mechanism through which estrogen signaling connects with the histamine system remains unclear. While it is assumed that complex interactions do exist among sex steroids and neural histamine in bringing about the facilitation of female sexual behaviors, still remains to be elucidated regarding the requirement of H1R in either estrogen action or subsequent progesterone action, or both. Future studies are needed to explore of a cooperative functional relationship between estrogen, progesterone and H1R in female VMN. It also remains to be determined if estrogen acts on H1R expression via ERα, how ERα induces H1R gene transcription, and whether this mechanism requires recruitment of other transcription factors.

In our experiments, we also found that progesterone significantly reduced the H1R expression level in the vl VMN in OVX female rats pretreated with estrogen. Since almost all ERα-expressing neurons coexpress estrogen-induced PR in the vl VMN region [42], H1R neurons in this region could be responsive to progesterone. However, our results provide no clear evidence for direct suppression of H1R expression by progesterone. Progesterone is a sex steroid hormone that is needed to facilitate female sexual behavior, acting as a downstream component of estrogen-induced sexual arousal [43], [44]. Progesterone affects neural activity via intracellular genomic effects and in a non-genomic manner by changing the binding affinity of the receptor by direct action on the receptor or altering the condition of the cell membrane [43], [45]. The relationships among estrogen, progesterone and histaminergic signaling are elaborate because progesterone plays a subordinate role in estrogen-ERα signaling. Expression of PR is strictly controlled by genomic regulation by ERα and is induced by estrogen; thus, the amplitude of the progesterone effect is primarily dependent on estrogen [30], [46]. Like estrogen, progesterone regulates a wide range of neurotransmitter related genes, including the neuropeptide Y1 receptor [47], mu-opioid receptors [48], glutamate receptors [49], GnRH [50], [51], and oxytocin [52]. Thus, both estrogen and progesterone act on the vl VMN to facilitate female sexual behavior, but in different ways. In our study, we found that H1R mRNA in the vl VMN was significantly induced in OVX female rats treated with EB, but that this effect was eliminated by subsequent progesterone treatment. These reciprocal effects of estrogen and progesterone have been described in the context of differential regulation by estrogen and progesterone of pre-proenkephalin mRNA expression in the female rat ARC [53]. In the vl VMN region, the reciprocal effects of estrogen and progesterone on vl VMN neurons are beginning to be revealed in female rat studies. Griffin and colleagues reported that estrogen and progesterone markedly regulate the dendritic structure of female VMN neurons, with estrogen-induced retraction that is reversed by progesterone treatment, suggesting a functional significance of this differential regulation in the vl VMN in female sexual behavior [54], [55]. However the underlying cellular mechanisms mediating the estrogen and opposite progesterone effects on vl VMN neurons require further study.

Further studies are also needed to determine the mechanism through which progesterone influences H1R expression in the VMN in association with estrogen signaling. Similarly, it has yet to be shown that regulation of H1R by sex steroids has functional consequences in modulating the sensitivity of particular neurons to estradiol and progesterone. However, it is likely that the sex steroid-induced changes in H1R observed in our experiments might have an impact on specific neural circuits, and consequently on sex related behavior. It is also possible that hormonal changes in H1R could be involved in other types of arousal functions, such as the histaminergic regulation of food intake. Possible involvement of neural histamine in the hypothalamic neural circuitry that regulates food intake has been suggested [56]–[60], but these studies have limited information on the anatomical definition (e.g., the regional specificity of the VMN and its subregions) and the relationship with the action of sex steroids. Thus, much work is needed to clarify the functional aspects of sex steroid-regulated H1R in the VMN.

In summary, the results of this study show the precise regional distribution of H1R in the vl VMN and indicate that H1R mRNA expression is induced by estrogen in female rats. Furthermore, we found an opposing effect of progesterone on estrogen-induced H1R expression in the vl VMN. Our findings are meaningful because there are few reports showing that H1R expression in the brain is significantly changed by sex steroids in adulthood, despite the many arousal-related physiological functions that are influenced by the neural histamine systems. These hormonal changes in H1R mRNA were observed in the vl VMN and not in the dm VMN. The regional differences in hormonal responsiveness provide further support for the notion that these two regions have different patterns of neural arousal in response to hormonal cues.

The central histamine system maintains arousal and increased receptor expression on the cell membrane should have a major impact on neural sensitivity to histamine. Based on our findings, we hypothesize that in estrous females, in which elevated estrogen primarily activates neural circuits controlling sexual arousal, estrogen can simultaneously amplify subordinate histamine signaling by increasing the proportion of vl VMN neurons that respond to histamine. These neurons are thought to undergo considerable neural excitation during estrogen-induced behavior, and thus enhancement of histamine signaling may be a powerful way to maintain a constitutively elevated status of neural excitability in the VMN.

## Supporting Information

File S1 **Verification of the specificity of the H1R antibody in H1RKO mouse.**
(DOCX)

File S2 **Verification of the specificity of the H1R antibody in recombinant cell lines.**
(DOCX)
