# Supplementary material for: Estrogenic Regulation of Histamine Receptor Subtype H1 Expression in the Ventromedial Nucleus of the Hypothalamus in Female Rats
Source: PLoS One. 2014 May 7;9(5):e96232. doi: 10.1371/journal.pone.0096232 (PMC4013143; doi:10.1371/journal.pone.0096232)
Supplement: File S1 — Verification of the specificity of the H1R antibody in H1RKO mouse. (DOCX) [file pone.0096232.s001.docx]

**Supporting Information (S1)**

**Verification of the specificity of the H1R antibody in a H1RKO mouse**

**Legends to Supplementary Figures**

**Supplementary Figure 1 (S1). Anti-H1R immunostaining in the H1R KO mouse and wild type mouse**

Representative photographs of H1R immunostaining in a wild type C57/B6 mouse (positive control, A and B) and H1R KO mouse (negative control, C and D). H1R immunoreactivity was detected in the vlVMN of the wild type mouse (A and B), but not in that of the H1R KO mouse (C and D). B and D are medium magnification images of neural groups in the mouse vl VMN (areas indicated with dotted lines in A and C, respectively). 3V, Third ventricle; Me, median eminence. Scale bars, 200 μm (A and C), 100 μm (B and D).

**Materials and Methods**

To confirm the specificity of the goat polyclonal antibody to H1R (LS-B1745; Life Span Bio Science, Inc.), immunostaining was carried out in a H1R KO mouse (Hrh1 KO/C75BL/6, male 10 weeks old, Oriental Bio Service, Inc.; Kyoto, Japan) [22] as a negative control and in a wild type mouse (C75BL/6, male 10 weeks old, Shimizu Laboratory Supplies Co.; Kyoto, Japan) as a positive control. Procedures for sampling of brain tissues, immunostaining and observations are described in the main text.

**Results**

Immunostaining revealed H1R immunoreactivity in the vlVMN of the wild type mouse (Supplemental Figure S1. A and B), but not in that of the H1RKO mouse (Supplemental Figure S1. C and D). These results show the reliability of the LS-B1745 antibody for specific recognition of H1R.

**(S1)**
