# Supplementary material for: Estrogenic Regulation of Histamine Receptor Subtype H1 Expression in the Ventromedial Nucleus of the Hypothalamus in Female Rats
Source: PLoS One. 2014 May 7;9(5):e96232. doi: 10.1371/journal.pone.0096232 (PMC4013143; doi:10.1371/journal.pone.0096232)
Supplement: File S2 — Verification of the specificity of the H1R antibody in recombinant cell lines. (DOCX) [file pone.0096232.s002.docx]

**Supporting Information (S2)**

**Verification of the specificity of the H1R antibody in recombinant cell lines**

**Legends to Supplementary Figures**

**Supplementary Figure S2. Anti-H1R immunostaining in recombinant cells overexpressing H1R-YFP or H2R-YFP**

Comparison of confocal microscopy images of immunofluorescence labeling of H1R between H1R-YFP COS-1 cells (positive control, A-C) and H2R-YFP COS-1 cells (negative control, D-F). A and D show YFP fluorescence, B and E show H1R immunofluorescence labeling (red), and C and F are merged images. In cells overexpressing H1R-YFP, H1R immunofluorescence labeling corresponded to the distribution of YFP-labeled H1R (A-C). Scale bar, 50 μm.

**Materials and Methods**

To investigate H1R-specific labeling of the antibody (LS-B1745), we also investigated immunostaining in recombinant COS-1 cell lines overexpressing H1R. For these experiments, we designed and constructed cell lines overexpressing H1R-YFP and or histamine receptor subtype 2 (H2R)-YFP as positive and negative controls, respectively.

**Plasmid construction**

Total RNA was extracted using ISOGEN (Nippon Gene, Toyama, Japan) from dissected hypothalamus tissue from female rat (SD, 10 weeks old) and reverse transcribed using ReverTra Ace (Toyobo, Osaka, Japan) and Oligo d(T)16 Primers. The complete rat H1R (D12800.1, Gene Bank) or H2R (S57565.1, Gene Bank) cDNA was isolated by PCR using specific primers designed for H1R (forward: TGA TCT TGT GTC TTC TTT TCT CCC, and reverse: GAA CGA ATG TGA ATC TTT TTG A) and H2R (forward: CCA AGA CCA AGA GGT GAA TGT AG, reverse: CTC CAC ACT TGG AGC TTC AAG G). The thermocycler protocol followed an initial denaturation cycle (5 min, 95°C); 33 cycles of denaturation (1 min, 95°C), annealing (1 min, 30 sec, 61°C), and extension (1 min, 72°C); and a final extension cycle (5 min, 72°C) terminating at 4°C. The amplified PCR DNA was isolated using gel electrophoresis, and then DNA fragments with the predicted size were extracted from the gel (QIAquick Gel Extraction Kit). The full-length coding region of H1R (1519 bp) or H2R (1075bp) was subcloned into a pGEM T Easy Vector (pGEM®-T Easy Vector Systems, Promega, Inc., Madison, WI, USA), and then each ligation mixture was transformed into highly competent *E. coli* DH5α cells. Transformants were selected on Luria Broth (LB) ampicillin-containing plates. Full-length H1R and H2R were isolated from the transformants using restriction digests and then fully sequenced. To create H1R-YFP and H2R-YFP constructs, plasmids containing the rat full-length H1R and H2R were digested using BglII and HincII restriction enzymes and ligated to the pEYFP-N1 vectors (BD Biosciences Clontech) using DNA Ligation Kit Mighty Mix (Takara, Tokyo, Japan). Each ligation mixture was transformed into *E. coli* DH5α cells, and transformed cells were selected on LB kanamycin-containing plates. Plasmid DNAs were isolated from correct constructs using NucleoBond^®^ Xtra Midi (Macherey Nagel, Düren**,** Germany).

**Cell Culture and Transfection**

COS-1 cells were maintained in a humidified atmosphere at 37°C with 5% CO_2_/95% air in Dulbecco's Modified Eagle's Medium (DMEM: Gibco, Palo Alto, CA). On the day before transfection, cells were reseeded on a poly-l-lysine-coated, 35-mm glass-bottom dish (Matsunami Glass, Inc., Ltd., Kishiwada, Japan) at an initial plating density of 3×10^4^ cells per well in 300 µl of medium for fluorescence microscopy observation. Plasmid DNA (250 ng) was transiently transfected into cells by a liposome-mediated method using LipofectAMINE PLUS (Invitrogen, Carlsbad, CA). Before analysis, the cells were washed with 400 or 1,000 µl of PBS and then cultured again in 400 µl of serum-free medium (OPTI MEM, Invitrogen).

**Immunohistochemistry**

COS-1 cells were fixed with 4% PFA in PBS, permeabilized with 2 ml of 2% BSA in 0.3% Triton X-100 in PBS for 60 min at room temperature, and then incubated with goat polyclonal antibody to H1R (LS-B1745; Life Span Bio Science, Inc.; 1:3,000 dilution) in blocking solution overnight at 4°C. Cells were then washed with PBS three times and incubated for 1.5 h with Alexa Fluor 568 anti-goat IgG (Molecular Probes, Inc., Eugene, OR). Immunostaining was observed with a confocal laser scanning microscope (LSM 510META, Carl Zeiss, Jena, Germany).

**Results**

Confocal microscopy imaging revealed that H1R-ir staining (red fluorescence) corresponded to the YFP-labeled H1R distribution (YFP fluorescence) in COS-1 cells overexpressing H1R (positive control, Supplemental Fig. 2. A-C). H1R-ir was not observed in COS-1 cells overexpressing H2R-YFP (Supplemental Fig. 2. D-F). These results also show that the LS-B1745 antibody recognizes H1R correctly and in a reliable manner.

**(S2)**
